# Extracorporeal Membrane Oxygenation (ECMO) and the Critical Cardiac Patient

**DOI:** 10.1007/s40472-017-0158-5

**Published:** 2017-07-10

**Authors:** David A. Baran

**Affiliations:** Advanced Heart Failure, Transplantation and MCS, Sentara Heart Hospital, 600 Gresham Drive, Norfolk, VA 23507 USA

**Keywords:** ECMO, Cardiogenic shock, Complications, Veno-arterial, Thoracic transplantation

## Abstract

**Purpose of Review:**

This review is to summarize the basics of veno-arterial (VA) extracorporeal membrane oxygenation (ECMO) as it is utilized for critically ill cardiac patients.

**Recent Findings:**

ECMO may be instituted in a variety of health care settings, from the emergency room to the operating room. The types of patients who may benefit from ECMO are reviewed in detail. The complications of ECMO are reviewed, including access-related issues and hematologic and neurologic problems. The principles of weaning of ECMO are described.

**Conclusion:**

Due to its versatility and relatively low cost, VA ECMO use is sharply increasing worldwide. It is important to select patients carefully for this mode of therapy as it can keep patients alive even in states of severe neurologic impairment or multiorgan failure. Short courses of ECMO may allow critically ill patients to be salvaged, but ultimately survival depends on resolution of the underlying problem or ability to transition to another more durable mode of cardiac support.

## Introduction

The treatment of heart failure has evolved exponentially over the last 30 years. Now, there are robust guidelines for the treatment of chronic systolic heart failure and numerous oral and intravenous medications which are approved to reduce mortality in this condition. The situation is far less clear in the setting of acute heart failure. The European Society of Cardiology [1••] and the American Heart Association/American College of Cardiology [2] both have guidelines which cover the topic of acute heart failure. The sickest patients are recommended for mechanical circulatory support therapy, and in particular, the use of the intra-aortic balloon pump is discouraged due to the failure of this therapy in post-infarct patients in the SHOCK II-IABP trial [3, 4]. The Society for Cardiac Angiography and Intervention released a consensus statement regarding the use of percutaneous mechanical circulatory support as well which helps fill in some of the gaps [5••].

Beyond the use of the intra-aortic balloon pump and other percutaneous mechanical circulatory support devices lies the use of extracorporeal membrane oxygenation (ECMO) therapy. At its core, this is the use of a miniaturized and simplified form of the venerable heart-lung bypass machine, and the use of this technology has increased in an extraordinary fashion over the last decade. There are many reviews of the use of ECMO in the literature, and the intent of this manuscript is not to duplicate the other works or encapsulate all their findings in an encyclopedic treatise [6–10]. Rather, this will serve as a review of the indications and practical aspects of this mode of therapy with liberal references for the reader to seek further detail where necessary.

ECMO has two predominant types: veno-arterial (VA) ECMO and veno-veno (VV) ECMO. Veno-veno ECMO is primarily utilized for patients with respiratory failure and not those with cardiac failure and therefore will not be discussed in this review, though the reader is directed to other reviews for this important topic [11–14]. In this review, ECMO is taken to mean veno-arterial ECMO.

## What is Veno-Arterial (VA) ECMO?

VA ECMO is a life support technique that involves withdrawing venous blood via an active pump and returning oxygenated blood into a major central artery. It functions as a parallel circuit to the patient’s heart and lung which perform the analogous functions in all humans. The first “heart-lung” machine was utilized by Dennis in 1951 to facilitate open-heart surgery at the University of Minnesota, and unfortunately, the patient died [15]. The device was enhanced by Gibbon and others and eventually allowed the surgical repair of cardiac defects [16].

All ECMO circuits involve four major components. There is an inflow cannula, which drains venous blood into the system, a pump which moves the blood through the system, an oxygenator which is a membrane system that functions analogous to the human lung, exchanging oxygen and carbon dioxide, and a return cannula which returns newly oxygenated blood to the systemic circulation. Additional components include a “blender” device which allows the specification of a particular percentage of oxygen versus room air components such as carbon dioxide and nitrogen, as well as a warming or cooling circuit which can control the temperature of the blood circulating through the system. In an operating room system, there is the addition of a large reservoir which allows the addition or subtraction of volume to the patient’s circulation as needed by the surgeon.

It is important to understand that the circuit and its components vary in biocompatibility and likelihood of sequestering platelets and depleting circulating elements necessary for the coagulation cascade. Typically, the operating room “bypass” machine is not well-tolerated for extended periods, and therefore, most operative reports specify the “bypass time” in minutes. On the other hand, modern VA ECMO circuits may be utilized for days at a time to support patients with fairly acceptable hematologic profiles. When dealing with a patient on VA ECMO support, it is important to understand which vessel and site are providing inflow to the pump, what kind of tubing and oxygenator are being utilized, and where the outflow is located. Additionally, it is important to know what diameter the inflow and outflow cannulas are, as this will determine the amount of blood that can flow in liters per minute. Cannulas are measured in “French” units where 3 French is equivalent to 1 mm in diameter.

Typically, for average adults, the venous (inflow) cannula is at least 21 French and the arterial outflow is 15–19 French. The clinician placing the cannulas needs to balance the desire for larger flows with the anatomic limitations of the patient and avoidance of ischemia due to obstruction of the circulation by catheters. Large venous cannulas are often well-tolerated, and the distal tip of the venous drainage cannula is often placed in the segment of inferior vena cava which is intra-hepatic, to avoid the risk of suction or entrapment of the wall of the right atrium or other vessel. The arterial cannula is typically placed in the femoral artery which can be a problem if the distal vessel is occluded. It is wise to use angiography or ultrasound to be certain that the large arterial return cannula is proximal to the bifurcation of the common femoral artery into the superficial femoral and profunda femoris. Another technique which is widely used is to place an antegrade “bypass limb” catheter into the superficial femoral artery at the time of retrograde large-bore cannula placement. This allows perfusion to the limb distal to the catheter, avoiding ischemic limb complications [17].

Once cannulas are placed, the operator must connect to the ECMO circuit, but with extreme care to avoid even small bubbles of air. Unlike an operating room bypass machine which has a large tub-like reservoir, which can allow the release of air bubbles, the peripheral ECMO circuit has no such release. Therefore, arterial air emboli could occur which can lead to coronary ischemia, ventricular fibrillation, as well as cerebrovascular accidents. Some systems such as the Cardiohelp from Maquet have an automatic “bubble detector” which will automatically stop pumping if air bubbles are detected.

Once air-free connections are made, the pump is activated and ECMO commences. The clinician is advised to start slowly and increase the flow gradually. Attempts to flow at higher rates may be limited by the phenomenon known as “chatter” marked by sudden drops in flow due to the inability to drain venous blood sufficiently fast to meet pump speed settings. The need for adequate venous drainage highlights the importance of using the largest feasible venous cannula when planning ECMO support. Similarly, an arterial cannula that is undersized will restrict inflow and lead to high circuit pressures.

States of profound vasodilation such as sepsis may limit rapid venous drainage and thus prevent adequate ECMO flows. Often times, volume resuscitation with crystalloid or blood transfusion may be necessary to avoid “chatter” and allow higher VA ECMO flows.

## Hemodynamic Effects of ECMO

ECMO has unique hemodynamic effects as compared to other modalities of cardiac support. First, venous drainage leads to reduced flow through the lungs and reduced strain on the right heart but the arterial afterload is increased commensurate with the ECMO flow. These hemodynamics are nicely illustrated in the analyses by Burkhoff et al. [18•] via pressure volume loops. The practical implication of this increased afterload is that the aortic valve may not open in cases of severe left ventricular dysfunction or asystole. This leads to elevations of left ventricular end diastolic pressure and can lead to frank pulmonary congestion. Particularly when VA ECMO support is prolonged, it is important to facilitate ejection of the left ventricle. This may be accomplished with inotropic therapy, placement of an intra-aortic balloon pump, or placement of an Impella LV–aorta pump [19–21]. Other options include creation of an atrial septostomy to decompress the left atrium.

When a patient is on VA ECMO, it is important to monitor the adequacy of saturation to the brain and also the ejection from the heart. In typical peripheral ECMO, arterial blood is returned to the femoral artery via short cannulas and therefore mixes with potentially less oxygenated blood which is delivered by the native heart. By checking pulse oximetry from the right hand, or arterial blood gas sampling from the right hand, it is possible to assess the oxygen level being delivered to the brain (via the right and left carotid arteries which are distal to the right subclavian artery. Ejection of the native heart can be assessed by serial echocardiography with attention to movement of the aortic valve but can also be accomplished by observing the pulsatility of an arterial pressure measurement catheter waveform. It is prudent to adjust the flow through the ECMO circuit on a periodic basis to facilitate ejection from the heart and avoidance of stasis and clotting either in the left ventricle or at the level of the aortic valve [22].

Another unusual complication of VA ECMO is the “harlequin syndrome” which occurs when deoxygenated blood ejected by the heart leads to cyanotic upper body with relatively well-oxygenated lower body (served by the oxygenated ECMO flow) [17]. This may require that the arterial inflow be relocated to the right axillary or subclavian artery which mixes the oxygenated ECMO blood with the output of the heart more proximally than that occurs with femoral arterial cannulation.

## Who is a Candidate for ECMO

In broadest terms, VA ECMO is most appropriate for patients who need both cardiac and lung support. The extreme case is a patient with cardiac arrest or refractory arrhythmias where any left-sided mechanical circulatory support device would be inadequate since both right and left ventricles are affected. In addition, patients with primary left ventricular failure with very elevated filling pressures are often ideal candidates for VA ECMO, particularly in the setting of severe pulmonary edema which may lead to profound impairments of oxygenation.

VA ECMO may be ideal in the setting of refractory ventricular arrhythmias. If it is impossible to terminate or control severe ventricular arrhythmias, placing the patient on VA ECMO is a relatively simple solution, as long as there is a path forward (such as ablation or definitive therapy such as a heart transplant or a total artificial heart placement) [23–26]. One issue to consider is whether the ventricles will eject in the setting of refractory arrhythmias. As mentioned previously, VA ECMO increases afterload and if left ventricular distention and stasis occurs, marked degrees of clot may occur despite the use of anticoagulation [22, 27, 28].

The other large advantage of ECMO is rapidity of deployment. Devices such as the TandemLife LA to Aortic pump (TandemHeart) can shunt blood from the left atrium to the femoral artery and may be used with an oxygenator unit spliced into the circuit if necessary. However, the necessity of a trans-septal puncture limits the rapidity of deployment as this requires specialized skill and usually fluoroscopy and imaging to safely place the large left atrial cannula. In experienced hands, VA ECMO cannulas may be placed at bedside even without fluoroscopy, and insertion times are quite short with growing experience. A recent case in France highlighted a mobile ECMO team who placed a patient on support on the floor of the famous Louvre Museum in Paris, France [29]. Emergency department use of ECMO is a growing practice but has a myriad of issues to consider, particularly due to the rapid decisions needed and the possibility that a patient may not have a viable strategy to wean from support [30–37].

VA ECMO may be lifesaving in cases of pulmonary vascular obstruction [38–41]. Patients with large pulmonary emboli are potential candidates for this mode of support, as long as a suitable venous drainage site can be located (i.e., no deep venous thrombosis obstructing). Since these patients are well anticoagulated during ECMO support, the technique is supportive but may also be therapeutic in nature. As well, patients who are waiting for lung transplant with severe pulmonary arterial hypertension may be well-supported temporarily by ECMO [42, 43].

Another category of patients who may be preferentially managed with VA ECMO are patients with severe biventricular cardiac failure. Rather than placing two assist devices which each have their attendant risks, it may be ideal to place a venous and arterial cannula and fully support the patient in this way. If this is temporary as may occur in severe myocarditis, ECMO may be a bridge to recovery, and weaning may occur a few days later. On the other hand, if a patient’s heart does not recover, VA ECMO may be used as a (risky) bridge to transplantation. This is a matter of extreme controversy in the heart transplant community as the outcomes of transplantation in patients on ECMO are inferior to those on lesser support [44]. Given the severe scarcity of organs, the utility of using ECMO to bridge patients to transplantation (as opposed to bridging to another type of durable mechanical circulatory support device) is hotly debated.

Lastly, as interventional cardiology and structural heart disease advance, there are increasing times when temporary support is necessary to treat a particular lesion. Examples include severe aortic stenosis with diminished left ventricular function undergoing transcatheter valve replacement, complex electrophysiologic ablation procedures which involve inducing arrhythmias which are not hemodynamically tolerated, and complex revascularization procedures with severe anatomical limitations.

## Where is ECMO Performed

In the early days of ECMO, the procedure was performed in an operating room, often with direct exposure of vessels by the surgical staff. The circuits were large and not very portable. In more recent times, the circuits have become smaller, and there has been a shift to percutaneous access as opposed to open access. In some hospitals, the cardiac catheterization lab is the preferred location given the ease of fluoroscopy and the availability of various wires and sheaths/dilators [45]. In other hospitals, the operating room is the preferred location, potentially with portable fluoroscopy or the use of trans-esophageal echocardiography for the placement of the long venous cannula.

Another aspect is that the ability to transport patients has improved with the smaller size of ECMO systems. This allows a team to place a patient on ECMO support at an outlying facility and then bring the patient back to the main hospital for further care. This was not possible with large bulky systems for ECMO but now is quite feasible [46]. As mentioned previously, emergency room ECMO [35, 36] and even emergent insertion in public places are possible with an experienced team [29]. Veno-arterial ECMO can be placed at the bedside without imaging in emergent circumstances, but one needs to be cautious about estimating the depth of insertion for the venous cannula based on a visual estimation of the distance to the right atrium from the skin insertion point. Each center needs to decide what resources they have to devote to this mode of therapy and where the preferred location for insertion will be. Often the choice depends on the type of inserting physician with surgeons preferring the operating room and cardiologists preferring the catheterization laboratory.

## When to Choose ECMO

In the “Who” section, we discussed the indications for ECMO, but this section is about the timing aspects of ECMO. In general, the risks of starting and maintaining an ECMO circuit need to be balanced against the risk of continuing without support. At the extremes, decisions are more straightforward. For example, a patient who is responding clinically to low-dose inotropes despite severely reduced ventricular function will probably incur more harm with ECMO than with continued conservative management. On the other hand, the patient with cardiac arrest that is refractory or one who is extremely ill and unstable may have benefit since the likelihood of death without advanced interventions approaches 100%.

It is critical to be sure the patient has a path forward prior to instituting ECMO support. For example, a patient with widely metastatic carcinoma who suffers a cardiac arrest but wants all aggressive care would still not be an appropriate ECMO candidate as the underlying disease is terminal. Patients with significant neurologic impairment particularly those with acute cerebral bleeds or cerebrovascular accidents are particularly poor candidates for ECMO given the reduced likelihood of meaningful recovery and the necessity of intense anticoagulation following ECMO. It is helpful to ask one’s self prior to starting ECMO whether there is a plan to solve the patient’s problems if ECMO cannot be weaned.

There are additional issues which should be considered when deciding when to institute ECMO. First, the success of VA ECMO is largely based on the ability to flow large volumes through the circuit, with the flow based on body surface area of the patient. If the patient has severe dehydration or marked vasodilation, the ability to rapidly draw 2–3 l per minute may be compromised. Similarly, if there are anatomic issues which prevent adequate cannula sizes from being utilized, the maximum flow through the circuit and hence the effect of the ECMO will be attenuated.

In cases where the decision to place ECMO is not clear, it is reasonable to place a Swan-Ganz catheter to measure hemodynamics and cardiac output. If a patient actually has hypovolemic or septic shock, these may be amenable to volume resuscitation at least initially. In addition, fluids can be provided to allow sufficient intravascular volume to utilize ECMO if cardiac status is quite compromised.

## Why is ECMO Use Expanding

ECMO therapy has a multitude of advantages over other modalities of advanced cardiac support. First, it is relatively inexpensive compared to percutaneous ventricular assist devices [47]. The major cost of a system is the pump head, and some such as the Rotaflow are less than $200. The other costs are related to the nursing care and the intensive care unit charges. In addition, perfusion personnel are required at the time of insertion and usually on a daily basis to check the circuit. Some areas mandate 24-h-7-day dedicated perfusionist coverage at the bedside which is a large cost. Secondly, placing ECMO is a skill familiar to all cardiac surgeons and increasing numbers of cardiologists are becoming facile with the placement of large-bore catheters. Lastly, the billing for care of ECMO patients is different than that of mechanical circulatory patients, and the complex issues which occur with these critically ill patients are reflected in the billing codes and number of resource value units (RVUs) in the program. All of these have resulted in a dramatic expansion of the use of ECMO for a variety of indications [48].

## How to Proceed Once ECMO is Started

Usually, most patients who are initiated on ECMO are in varying states of metabolic disarray, and some may be in peri-cardiac arrest setting. It is reasonable to check labs and blood gas analysis frequently in the first few hours with a goal of correcting acid-base status and electrolyte balance. If neurologic status is uncertain, it is appropriate to withhold sedation and paralytic agents to allow an assessment to be made. Usually these patients are not ideal to move from the ICU, and thus, a focus on bedside evaluations is made, including chest X-rays and ultrasound if needed to answer a clinical question. Electroencephalography may be helpful if seizure activity is suspected.

Once the patient is semi-stable, efforts should be directed towards a plan to minimize the duration of support. Planning for the next step, whether that is eventual withdrawal of ECMO or transition to another form of support such as mechanical circulatory support, is paramount in importance. It is also critically important to have team meetings daily among all the disciplines involved with the care of the patient, as well as updating the family of the patient.

ECMO should be thought of similarly to cardiopulmonary resuscitation, with a “code leader” and the expectation that the duration of support is in the discretion of the medical team. Since ECMO provides complete support of both heart and lungs, it is possible to maintain life even in cases when there is devastating neurologic injury or multiorgan failure which prevents weaning of support. For this reason, we have learned the importance of involvement from the earliest possible time of social work and palliative care, to assist in the interface of the medical team and the family. Local laws need to be consulted on the ability to withdraw futile ECMO support in the face of opposition of relatives of the patient, but it is far preferable to set expectations prior to the institution of support if possible so that there is trust in the medical team and respect for the ultimate authority of the ECMO team to make the best choices. In difficult situations, consultation with the Ethics Committee may be quite helpful [49•, 50, 51].

The process of weaning of ECMO involves reducing the flow through the device and assessing the response [52]. Anticoagulation must be sufficient so that the circuit does not develop clots during the time of lower flows (1–2 l per minute). Echocardiography or trans-esophageal echocardiography can be extremely helpful in guiding this process, particularly if recovery of the heart may be expected such as in cases of myocarditis or sepsis-induced cardiac dysfunction or post-cardiac arrest. It is important not to reduce the oxygenation fraction of the circuit as is commonly done with veno-veno ECMO as this will result in increasing levels of right-to-left shunting (venous blood returned to the arterial circulation without full oxygenation) and undesirable results.

Another bedside sign of recovery is the degree of pulsatility. Often the arterial line tracing on ECMO is relatively flat with little pulsation indicating weak if any opening of the aortic valve. As the heart recovers (perhaps facilitated with inotropes or simply correction of acidosis and metabolic disarray), the arterial trace will develop a larger pulse pressure which is a valuable sign. As well, as discussed previously, there is a danger of cardiac thrombosis if the heart does not eject blood and we strive to maintain flow at a level allowing at least some ejection of blood, even if the ECMO flow needs to be lowered to accomplish this goal. In addition, a lack of ejection will result in pulmonary congestion, which makes weaning of support quite problematic. Intra-aortic balloon pumps, Impella pumps (Abiomed, Danvers, MA), and even atrial septostomy have been used to address this issue.

The usual length of support is less than 7–10 days. ECMO is best used as an inherently temporary intervention and transitioned to another strategy within this time period. Deciding on which intervention is appropriate involves the collective efforts of the cardiothoracic surgeon, advanced heart failure/transplant program, critical care medicine, perfusion, pulmonary medicine, as well as the social work and palliative care teams.

## Complications of ECMO

A full discussion of the complications of ECMO is beyond the scope of this manuscript [53, 54, 55]. However, complications can be divided broadly into categories: access-related, hematologic, and neurologic.

Access-related issues begin at the time of large-bore cannula placement, and while issues may affect the venous cannula, almost always the issues are with the arterial catheter. An understanding of the size of the vessel that the cannula is placed in is essential, and yet often there is insufficient data to guide decisions, particularly in emergent percutaneous placement of ECMO. Distal ischemia can lead to arterial thrombosis and gangrene, and for this reason, a close relationship with the vascular surgery team is essential to avoid delays in diagnosis and treatment of these devastating complications. A useful strategy is placement of a small distal perfusion catheter to bypass the area of obstruction from the ECMO arterial cannula [56–60]. Alternatively, placement of a surgical graft avoids the risk of obstruction of the artery but requires open surgical placement which is not always available and a patient may not be sufficiently stable to tolerate a trip to the operating room for this intervention.

Hematologic issues with ECMO are quite common since the therapy involved continuous flow through plastic tubing as well as an oxygenator with a large surface area [61–63]. Anticoagulation is typically accomplished with short-acting agents such as heparin or direct thrombin inhibitors, with monitoring by activated clotting time or partial thromboplastin time. In general, activated clotting time is maintained above 200 s particularly early following ECMO initiation, but the goal needs to be adjusted by the team depending on bleeding, clot formation in the oxygenator, and the presence of other issues such as trauma. Clotting can occur in the circuit or may manifest as thromboembolic events including cerebrovascular, renal, or splenic infarcts. Bleeding can occur at any site but most commonly at the site of access placement, particularly if the insertion was traumatic. It is paramount to ensure that the access site has proper hemostasis when insertion occurs as it is easier to place purse string sutures and other maneuvers when the field is sterile than to rely on pressure bandages or other ineffective modalities.

The most feared bleeding complication is cerebral hemorrhage, and this may be fatal when it occurs even if anticoagulation is discontinued. If possible, it is reasonable to obtain computed tomography of the head prior to institution of ECMO if there has been any period of cardiac arrest, but this is often not possible. A recent review from the ELSO (Extracorporeal Life Support Organization) Registry estimated the incidence of neurologic complications at 15.1% (brain death 7.9%, cerebral infarction 3.6%, seizures 1.8%, and cerebral hemorrhage 1.8%). Notably, mortality was significantly higher in those with neurologic complications versus those without (89 vs 57%, *p* < 0.001). In a multivariable model, age, pre-extracorporeal membrane oxygenation cardiac arrest, the use of inotropes on extracorporeal membrane oxygenation, and post-extracorporeal membrane oxygenation hypoglycemia were shown to be associated with CNS complications [64].

## Conclusion

ECMO is widely used as a technique to treat critically ill patients with cardiogenic shock and cardiac arrest. It can be placed in a variety of settings and by a variety of physicians. Advances in technology have led to reduced costs, and ECMO may be less expensive than some percutaneous mechanical support devices, which enhances its utility. The use of ECMO is likely to climb, and therefore, knowledge of the management and weaning of patients is increasingly important. Complications are not infrequent and need to be balanced against the potential benefits of this mode of therapy.
